# AI carbon footprint: The non-negligible hidden emission source

**DOI:** 10.1016/j.eehl.2025.100197

**Published:** 2025-11-03

**Authors:** Chao He, Fang Yue, Lanchun Li, Yun Tang, Qian Wu, Wei Chen

**Affiliations:** a National Science Library (Wuhan), Chinese Academy of Sciences, Wuhan 430071, China; b Hubei Key Laboratory of Big Data in Science and Technology, Wuhan 430071, China; c School of Resource and Environmental Sciences, Wuhan University, Wuhan 430079, China; d Department of Information Resources Management, School of Economics and Management, University of Chinese Academy of Sciences, Beijing 100190, China; e Institutes of Science and Development, Chinese Academy of Sciences, Beijing 100190, China

**The rapid advancement of artificial intelligence (AI) is driving global digitalization, but its soaring energy demands and carbon emissions present pressing environmental challenges. To balance AI's growth with sustainability goals, this paper proposes a Dual-Circular AI Sustainability (D-CAIS) framework, emphasizing both internal energy optimization and external emission reduction across sectors.**

The rapid advancement and deployment of artificial intelligence (AI) technologies are propelling a new wave of digital transformation across industries [1,2]. Yet, alongside its transformative potential, AI development generates significant environmental externalities, particularly through intensive energy consumption and associated carbon emissions [3]. Large-scale models such as GPT-4 and Gemini Ultra require millions of kilowatt-hours during training, releasing tens of thousands of tons of CO_2_ equivalent (CO_2_eq) [4]. These environmental burdens are further magnified by the global expansion of data centers, many of which remain reliant on fossil-based electricity [5]. Without targeted mitigation, the continued scaling of AI may jeopardize global carbon neutrality, aggravate energy inequality, and intensify ecological stress [6].

Although some governments have introduced policies to improve the energy efficiency of the AI industry [7], the sector's carbon footprint is still growing exponentially, becoming a hidden yet critical emission source in global climate governance. High energy demand and carbon output not only worsen the climate crisis [8], but also challenge resource allocation and social equity [9]. Operational emissions account for about 90% of data centers' life cycle impact, and if current trajectories persist, data storage services alone could contribute 14% of global carbon emissions by 2040 [10]. Meanwhile, the global race for computing power has increasingly become a competition for energy resources, deepening regional imbalances in electricity supply, particularly in fossil fuel-dependent regions, and raising carbon tax burdens [4].

In response, the notion of “Green AI” has emerged, promoting environmentally conscious approaches to algorithm design and system deployment [11]. Efforts in carbon accounting and hardware optimization have provided critical insights, yet most remain confined to technical dimensions—emphasizing metrics, infrastructure, or efficiency—without establishing an integrated governance framework that links AI development with sustainability goals. Similarly, the call to employ AI for industrial decarbonization remains largely conceptual, often overlooking the feedback dynamics required for systemic transformation [12].

Moreover, critical tensions persist in the literature. Scholars remain divided over whether AI is a net environmental threat or a key enabler of climate solutions. Some have proposed frameworks such as “Green-by-Design” or “AI-for-Sustainability”, but these often remain fragmented, lacking scalability and operability [13]. Importantly, few frameworks consider dual-source carbon emissions, both direct and indirect, in a single integrated structure. To bridge these gaps, we propose the D-CAIS framework, which integrates internal (efficiency-driven) and external (application-driven) emission reduction cycles, linked by performance metrics (e.g., CPUE) and policy tools. D-CAIS provides a scalable path to align AI development with climate goals.

## 1 D-CAIS framework and the mechanism pathways

**Fig. 1:**
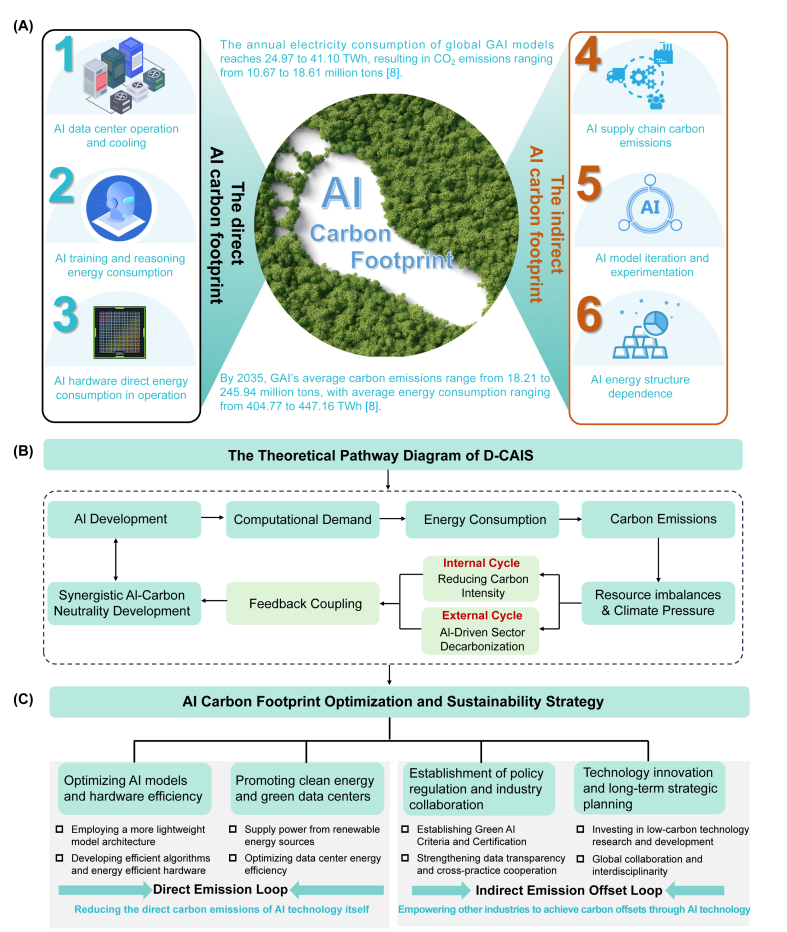
The primary sources of AI's carbon footprint (A), the theoretical framework of D-CAIS (B), and corresponding sustainable emission reduction strategies for AI's carbon footprint (C). Panel (A) elaborates on three major direct emission sources (left column) and three indirect sources (right column) contributing to the carbon footprint in current AI development. Additionally, two specific cases are cited (in light blue text) to highlight the urgency of managing AI's carbon emissions. Panel (B) depicts the causal process of AI carbon emissions and their feedback coupling with the ecological environment. Panel (C) details the dual-cycle strategy for sustainable carbon emission reduction constructed on the basis of (B), which aims to reduce direct carbon emissions from AI technology itself (internal cycle) and achieve carbon offset by empowering other industries through AI technology (external cycle).

The carbon footprint of AI stems from both direct sources, such as training and deploying large-scale models, data center cooling, and GPU manufacturing, as well as indirect emissions from supply chains and iterative model optimization (Fig. 1A). In response, we propose the D-CAIS Framework, which outlines a structured pathway to mitigate AI's environmental impact through a dual-governance mechanism. This comprises an Internal Cycle focused on technology-side decarbonization (e.g., improving computational efficiency and reducing direct emissions) and an External Cycle that promotes AI-enabled carbon reduction across industries, thereby creating an indirect emission offset loop. The framework incorporates theoretical feedback loops to capture the dynamic co-evolution between AI innovation and environmental regulation, forming a recursive structure aimed at sustainable AI development (Fig. 1B).

## 2 Internal and external cycle strategy for AI carbon footprint

The internal cycle improves AI energy efficiency through lightweight models, low-energy algorithms, and optimized processors. Transitioning to renewables and upgrading data center infrastructure are key priorities. For example, Alibaba's AI-driven cooling system reduces energy use by over 20%, cutting emissions and costs [14]. These integrated efforts align AI infrastructure with low-carbon goals. The external cycle empowers high-emission industries (e.g., energy, transport) through AI-driven optimization. Initiatives like Tencent's “Carbon LIVE” facilitate cross-sector knowledge sharing, while global collaboration fosters tech diffusion and interoperability. Tools like CPUE and green certifications link both cycles, reinforcing sustainable transitions across the innovation-empowerment-feedback chain (Fig. 1C).

## 3 Results and policy recommendations

The rapid expansion of AI has intensified energy demand and carbon emissions, challenging global climate goals. We propose the D-CAIS framework, which integrates two pathways: an internal cycle focused on reducing emissions through lightweight models, energy-efficient hardware, and renewable power, and an external cycle that applies AI to decarbonize sectors such as manufacturing, construction, and transportation via process optimization and cross-sector knowledge transfer. These cycles are mutually reinforcing, aligning technological diffusion with emission mitigation. Realizing this potential requires coordinated policies that standardize carbon accounting across AI lifecycles, integrate green AI metrics such as CPUE into infrastructure strategies, and strengthen international collaboration for equitable access to sustainable policy instruments, such as tax credits, carbon pricing, and green finance, to accelerate the adoption of renewable-powered data centers.

## 4 Challenges and future directions

Despite the promise of the D-CAIS framework, several challenges remain. Limited data availability and transparency constrain accurate assessments of AI's energy use and emissions, impeding effective policy design. Carbon inequality and technological disparities between developed and developing nations risk deepening both digital and environmental inequities, underscoring the need for international support mechanisms. Moreover, the slow adaptation of policy to rapidly evolving technologies highlights the urgency of anticipatory governance. Future research and policy should prioritize two directions: integrating AI systems with renewable energy infrastructure to leverage AI for energy optimization and management, and establishing global standards for AI carbon auditing to ensure interoperability of sustainability metrics and foster a unified approach to low-carbon AI development. Aligning AI's trajectory with climate imperatives is essential to ensure that its expansion advances not only technological progress but also sustainable development.

## CRediT authorship contribution statement

**Chao He:** Writing – original draft. **Fang Yue:** Resources. **Lanchun Li:** Data curation. **Yun Tang:** Investigation. **Qian Wu:** Validation, Software. **Wei Chen:** Funding acquisition.

## Declaration of competing interest

The authors declare that they have no known competing financial interests or personal relationships that could have appeared to influence the work reported in this paper.
